# Comprehensive environmental impact assessment and irrigation wastewater suitability of the Arab El-Madabegh wastewater treatment plant, ASSIUT CITY, EGYPT

**DOI:** 10.1371/journal.pone.0297556

**Published:** 2024-02-29

**Authors:** Ahmed M. El-Feky, Mohamed Saber, Mahmoud M. Abd-el-Kader, Sameh A. Kantoush, Tetsuya Sumi, Faisal Alfaisal, Amal Abdelhaleem

**Affiliations:** 1 Agricultural Engineering Department, College of Food and Agriculture Sciences, King Saud University, Riyadh, Saudi Arabia; 2 Water Resources Research Center, DPRI, Kyoto University, Goka-sho, Uji City, Kyoto, Japan; 3 Department of Civil Engineering, College of Engineering, King Saud University, Riyadh, Saudi Arabia; 4 Egypt-Japan University of Science and Technology, New Borg El-Arab City, Alexandria, Egypt; Siksha O Anusandhan University Institute of Technical Education and Research, INDIA

## Abstract

The presence of a wastewater treatment plant in the Arab El-Madabegh region, which discharges excessive amounts of raw effluent toward the nearby farming fields, is the area’s main issue. Examining the harmful implications of raw effluent releases on groundwater quality, determining if treated wastewater effluent complies with regulations for discharge into the aquatic environment, and assessing irrigation appropriateness by the effluent are the main goals of this work. In order to accomplish these targets, twelve treated effluent samples from the Arab El-Madabegh wastewater treatment plant were gathered every two weeks starting in January 2012 and finishing in June 2012. They were tested to determine pH, Total Dissolved Solids (TDS), Total Suspended Solids (TSS), Temperature (Temp), Conductivity (EC), Turbidity (Turb.), Chemical Oxygen Demand (COD), Biological Oxygen Demand (BOD), Total Organic Carbon (TOC), NO_3_^-^, SO_4_^2-^, Cl^-^, Ca^2+^, PO_4_^3-^, HCO_3_^-^, Na^+^, Mg^2+^, and heavy metals such as (Fe, Mn, K^+^, Cr, Pb, Zn, Ni, Cu, and Cd). The outcomes revealed that all Egyptian and Food and Agricultural Organization (FAO) standards for unrestricted irrigation were met by the treated effluents, except for COD, which exceeded than the Egyptian allowed limit. The evaluation indices of the effluent’s EC, SAR, PI, MR, and MH were in the low-risk category according to indicators of water quality for irrigation, nevertheless, The SSP and RSC both showed slightly higher values (67.9% and 2.76, respectively). As well, The average values of heavy metals in treated wastewater effluent were found to be below permitted limits, with the exception of lead and phosphate, which exceeded permissible limits in Egypt. The environmental sustainability (ecological friendliness) of reusing and recycling tertiary treated wastewater can be achieved in agriculture to reduce the adverse impacts on the aquatic environment.

## 1 Introduction

Our awareness of the environment is increasing with the growing human population. With each new inhabitant on the planet, it becomes crucial to ensure a reliable supply of clean energy, water, soil, and air. To safeguard resources for future generations, it is evident that maintaining the cleanliness of our resources. Globally, the availability of fresh water for irrigation is gradually decreasing [1]. Given the rising water demands, managing the region’s water resources will remain extremely challenging due to the scarcity of surface and groundwater [2]. Reusing treated wastewater for irrigation present a viable means of completing the water cycle in the agricultural and industrial sectors. Wastewater reclamation could produce treated effluents that farms with limited access to irrigation water could use, thereby alleviating pressure on conventional supplies, especially during the drier seasons [3]. Consequently, priority should be given to utilizing cutting-edge technology for developing new sources, such as wastewater reuse. Reusing treated effluent from municipal wastewater treatment plants (WWTP), typically released into the environment, is gaining popularity as a sustainable water supply in recent years [4]. In areas with limited freshwater, extensive treatment or reuse of municipal wastewater is crucial due to the return flow of 75–85% of each cubic meter used [5]. Over the past 20 years, reusing municipal wastewater has emerged as a significant alternative water supply and a practical way to provide the agricultural sector with water in several nations [6–8].

Environmental sanitation is a major issue that needs to be addressed as our societies develop. One of the elements that are essential for both human and economic development is adequate sanitation, especially for poor urban residents in developing countries today. 40% of the urban population in low-income nations remained unserved despite significant investments made during the water and sanitation decade [9, 10]. There have been significant improvements in household sanitation access since 2000; with 163 million people in Sub-Saharan Africa now have access to fundamental sanitation services. Even though, 709 million people still lack access to basic sanitation services [11]. By 2030, everyone has equitable access to safe, affordable drinking water, sanitation, and hygiene, according to Sustainable Development Goal 6 (SDG) [12].

Several millions acres are now irrigated with wastewater discharges as a result of China’s tremendous development of wastewater use in agriculture over the past few decades [4]. The utilization of treated municipal wastewater for irrigation is considered an eco-friendly wastewater disposal method compared to direct disposal into surface or groundwater [13, 14]. The quality of wastewater effluent is a crucial factor in wastewater reuse for agricultural applications, as it must meet standards to safeguard the environment, human health, and be suitable for soil and plants [15]. Although precautions are necessary to prevent adverse impacts on human health and the environment, wastewater reuse in agriculture is commonly accepted for agronomic and economic reasons [16–20]. Furthermore, the option of wastewater reuse is becoming necessary due to the worsening effects of climate change, leading to droughts and water shortage. Stricter rules governing the discharge of wastewater effluent have contributed to improving the water quality [19].

The utilization of wastewater effluent for irrigation is also a relatively cheap method of wastewater disposal, in addition to providing the soil with nutrients and organic matter [21]. However, it is advisable to refrain from irrigating edible vegetable crops with wastewater effluent. Instead, Other plants, including woody trees that can act as a windbreak and plants that produce energy, may be irrigated with wastewater [22]. It is necessary to establish Egyptian guidelines for the reuse of these waters in agriculture for instance, all treated wastewater effluents must be reused according to national rules in Jordan and Saudi Arabia.

Treated wastewater (TWW) reuse increased from 0.2 m^3^ to 2.0 billion m^3^ by 2017 and is expected to reach 2.4 billion by 2025 based on Egypt’s water policy objectives [23]. Egypt’s Ministry of Water Resources and Irrigation has set water quality standards for treated wastewater to protect freshwater watercourses, addressing conditions for the indirect reuse of wastewater in countries like Egypt, Cyprus, Italy, Spain, and Cyprus. It is currently possible to irrigate woodland to produce wood and safeguard the environment. The recommended concentrations of heavy metals are 0.5, 0.1, 0.05, 0.01, and 0.1 mg/L.

A study conducted in the northern part of El-Fayoum Governorate in Egypt revealed that soils irrigated with wastewater for more than 30 years contained significantly higher levels of heavy metals compared to fields irrigated with Nile water. The researchers noted that prolonged cultivation with raw municipal sewage led to increased levels of lead (Pb), particularly, in clayey soils compared to sandy soils. The study also suggests the necessity for developing integrated wastewater treatment facilities, regulating industrial operations, and managing urban growth and long-term agricultural practices that involve wastewater irrigation [24]. Crop samples taken from Egypt’s El-Saff wastewater canal in southern Giza governorate indicated that most metal concentrations were within the guidelines set by the Food and Agriculture Organization (FAO) and FAO/World Health Organization (WHO). The highest quantities of zinc (Zn) and copper (Cu) were found in fava beans, garlic, peppermint, and fava bean and onion. additionally, the majority of metal concentrations remained below health-standard limits, and the health risk index suggested minimal risk for both people and animals [25]. The physicochemical properties were also compared with WHO and Egyptian standards in Greater Cairo, Egypt [26]. The levels of BOD_5_, COD, TSS, and NH_4_^+^ were determined to exceed allowable limits. Multivariate statistical analysis indicated a decline in water quality during the preceding three decades. Within the framework of Bahr El-Baqar, Bilbies, and El-Qalyubia in Egypt, agricultural drainage water samples were collected over a year to assess their suitability for irrigation. The water was classified as high salinity low sodicity (C3S1), indicating it may not be suitable for soils with poor drainage. However, with effective salinity and drainage management, the water could potentially be used for agriculture. Water from Bahr El-Baqar, classified as non-sodic with low salinity, is categorized as C2S0, while water from El-Qalyubia, classified as non-sodic and has normal salinity, is categorized as C1S0. Crops that are not susceptible to salt and can be cultivated in these waters. In the context of Iraq, significant amounts of Sulaimani City effluent in industrial areas have been found, leading to direct dumping into the Tanjero River. A survey of 31 locations revealed that 48% of the impacted areas had chronic illnesses, 10% had diarrhea, 10% had typhoid, 10% had skin conditions, 6% had cancer. The most common chronic illnesses were diabetes and hypertension [27].

Shakir et al. [28] studied the sustainability of the Al-Rustamia wastewater treatment plant in Iraq and its potential for treated wastewater use in irrigation. They identified high salinity and chloride hazard in the wastewater, making it unsuitable for summer and fall irrigation. The researchers recommended the construction of two wastewater treatment plants and implementing of on-site sanitary treatment. In another study assessing the environmental and health implications of using treated wastewater for irrigation [28]. The data revealed minimal pollution, with an average comprehensive pollution index of 0.69 and an Organic pollution index ranging from 1.29–1.60. Despite high salinity in summer and fall and limited irrigation at some sites due to potential chloride hazards, the wastewater was generally deemed suitable for irrigation [29]. Osman et al. [30] evaluated the hazard of heavy metal contamination in crops and vegetables cultivated in freshwater (FW) and treated wastewater (TWW) irrigation locations. The findings indicated that the heavy metal levels in both TWW and FW are below Egyptian and FAO guidelines. TWW-irrigated plants had higher adult and child heavy metal transfer factor values, as well as higher chronic daily intake of metals and health hazard risk values. Strategies to reduce heavy metal levels included preventing overuse of pesticides and fertilizers, along with ongoing market surveillance. Moussaoui et al. [31] assessed various water quality variables at the wastewater treatment plant (WWTP) in Ain Sefra, southwest Algeria. Results revealed good organic material degradation, efficient biological processes, and excellent nitrate removal. Although ammonia levels exceeding acceptable limits, phosphate levels met Algerian requirements for irrigation and FAO regulations. The Ain Sefra WWTP was deemed appropriate for agricultural use, with nitrate levels below allowable limits, affirming efforts to prevent groundwater pollution. Badr et al. [32] examined the quality of irrigation water in Al-Ahsa Oasis using 104 water samples. They discovered that treated wastewater combined with groundwater is suitable for irrigation with Higher levels of TDS, cations, and anions associated with spatial changes in water quality. According to the irrigation water quality indices, 4.1% and 62.1% of the samples were consider acceptable and adequate for irrigation, respectively. A 2020 investigation assessed 12 irrigation water samples and soil profiles and discovered that long-term wastewater irrigation resulted in higher heavy metal concentrations in soils than fresh water. Alnaimy et al. [24] found that continued cultivation with raw municipal effluent resulted in higher Pb contents particularly, in clayey soils, emphasizing the need for long-term agricultural management solutions involving wastewater for irrigation.The most common water quality variables of concern in municipal wastewater treatment systems are Biological Oxygen Demand (BOD), Chemical Oxygen Demand (COD), Dissolved Oxygen (DO), suspended solids, nitrate, nitrite, ammonia, phosphate, salinity, various other nutrients and trace metals [33–35]. Heavy metal, being non-degradable and accumulating in the food chain, are enduring pollutants in wastewater, posing potential risks to human health and causing ecological disturbances [4]. The Tertiary Wastewater Treatment Plant at Buraidah City consistently met the high standards set by ministry of electricity and agriculture of Saudi Arabia for unrestricted irrigation [36].

### 1.1 Problem statement

**Fig 1:**
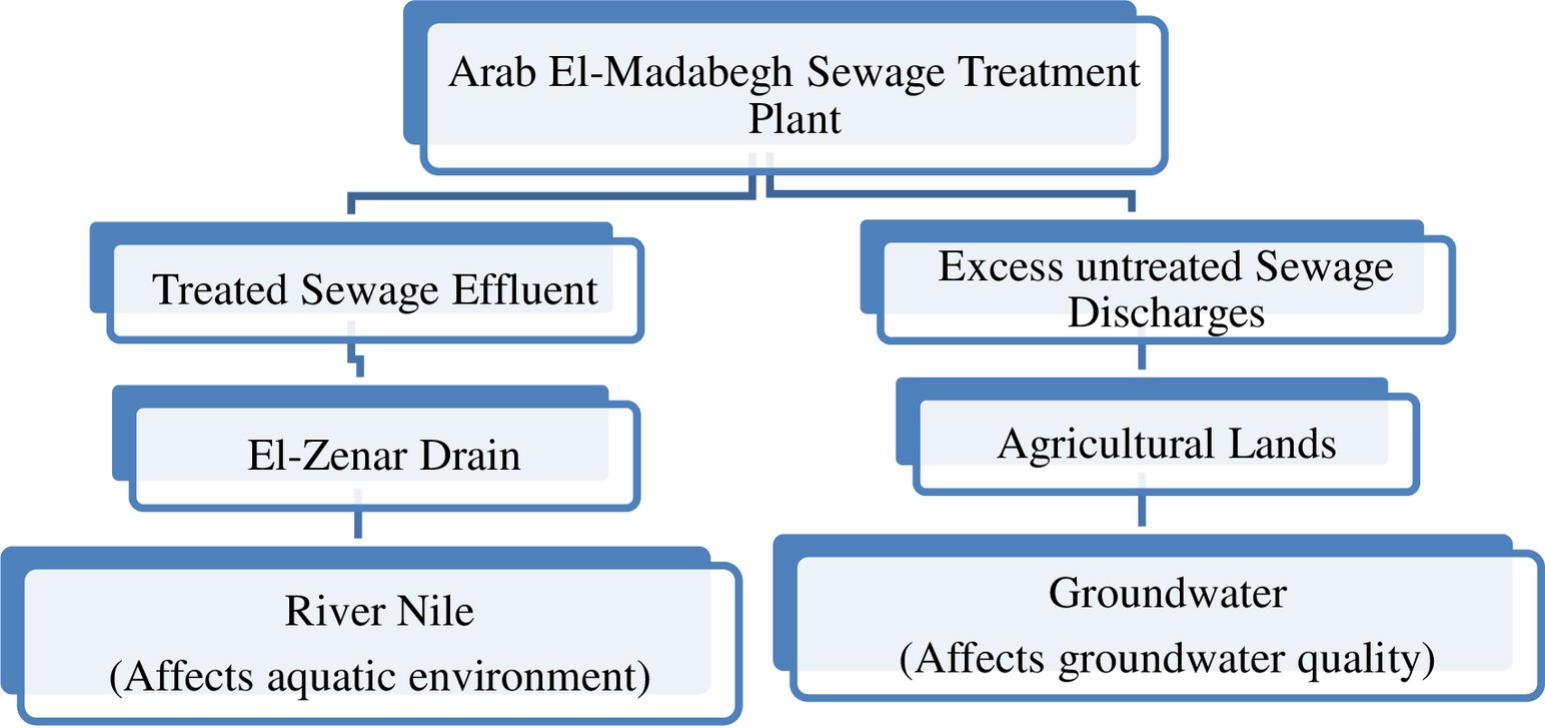
Graphical representations showing main problem of Arab El- Madabegh area.

The majority of TWW at Arab El-Madabegh is discharged into the El-Zenar drain. approximately 65000 m^3^/day of treated wastewater effluent. The El-Zenar drain contributes to the freshwater areas impacted by wastewater and ultimately flows into the River Nile. The stream’s impact on water quality and biological resources leads to a loss of biodiversity [37]. It is common knowledge that wastewater plants treat wastewater in stages, reducing the amount of suspended solids, organic matter and nutrients to minimize contaminant strength in the final effluent that was brought to the plant [38]. Fig 1 show the graphical representations of the major issue Arab El- Madabegh area.

**Fig 2:**
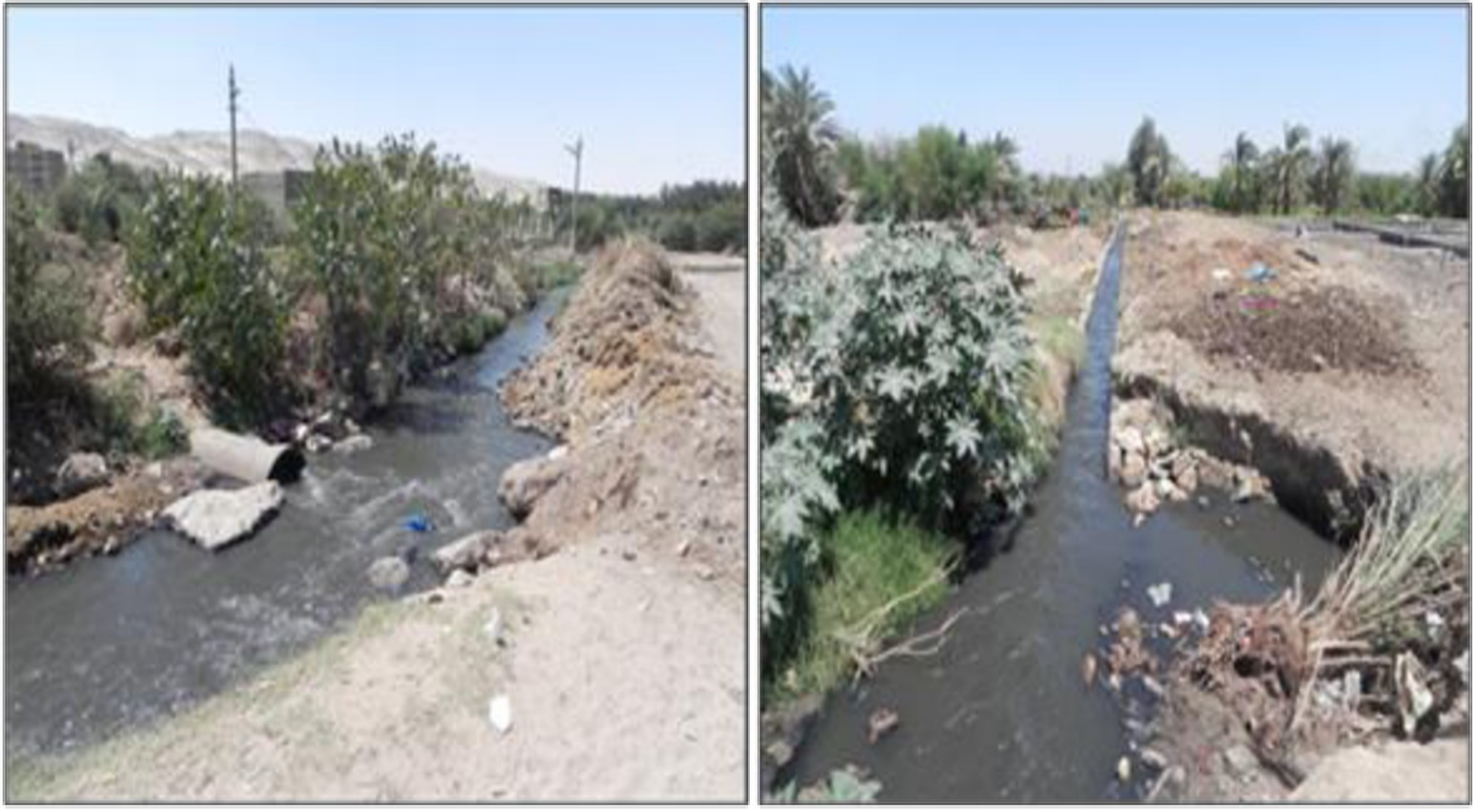
On the left is showing a By-pass pipe of the plant and on the right is showing an untreated wastewater discharges passing through the agricultural area.

A notable concern is the presence of three WWTP in the Arab El Madabegh area of the Assiut Governorate of Egypt. Two of these plants are secondary treatment facilities with design capacities of 20,000 m^3^/day and 30,000 m^3^/day, while one is a tertiary treatment facility with a design capacity of 70,000 m^3^/day. The tertiary treatment facility is operating beyond its designed capacity because all contaminated wastewater is discharged into it. Consequently, a bypass pipe discharges around 55,000 m^3^/day of untreated wastewater onto the nearby agricultural area (Fig 2).

The potential for groundwater pollution in Arab El-Madabegh is increasing dramatically in the coming days, along with a massive rise in raw wastewater flows, suggesting that the situation will become more severe and inescapable. The TWW effluent is pumped directly via a pipeline to the El-Zenar drain, which empties into the Nile. In fact, no studies have been conducted on the plant to evaluate the impact of the end effluent released into the surface and groundwater in Arab El-Madabegh. Aquatic ecosystems are greatly impacted by environmental pressure as they are the typical receivers for domestic effluents.

The effectiveness of the Arab El-Madabegh wastewater treatment plant’s treatment processes are examined in this study, as well as the environmental level indicators in the treated wastewater effluent. It also assesses the impact of the plant’s treated wastewater discharges on the aquatic ecosystem by contrasting these indicator values with the Egyptian regulations for the preservation of the aquatic ecosystem. Moreover, it evaluates its suitability as an unconventional source of water for irrigation. The unique aspect of these investigations is the comprehensive environmental assessment of the Arab El-Madabegh WWTP and its suitability for irrigation based on physical, chemical, and heavy metal characteristics of treated wastewater effluents, alongside indicators for evaluating the effectiveness of the effluent for irrigation.

## 2 Materials and methods

### 2.1 Study area

**Fig 3:**
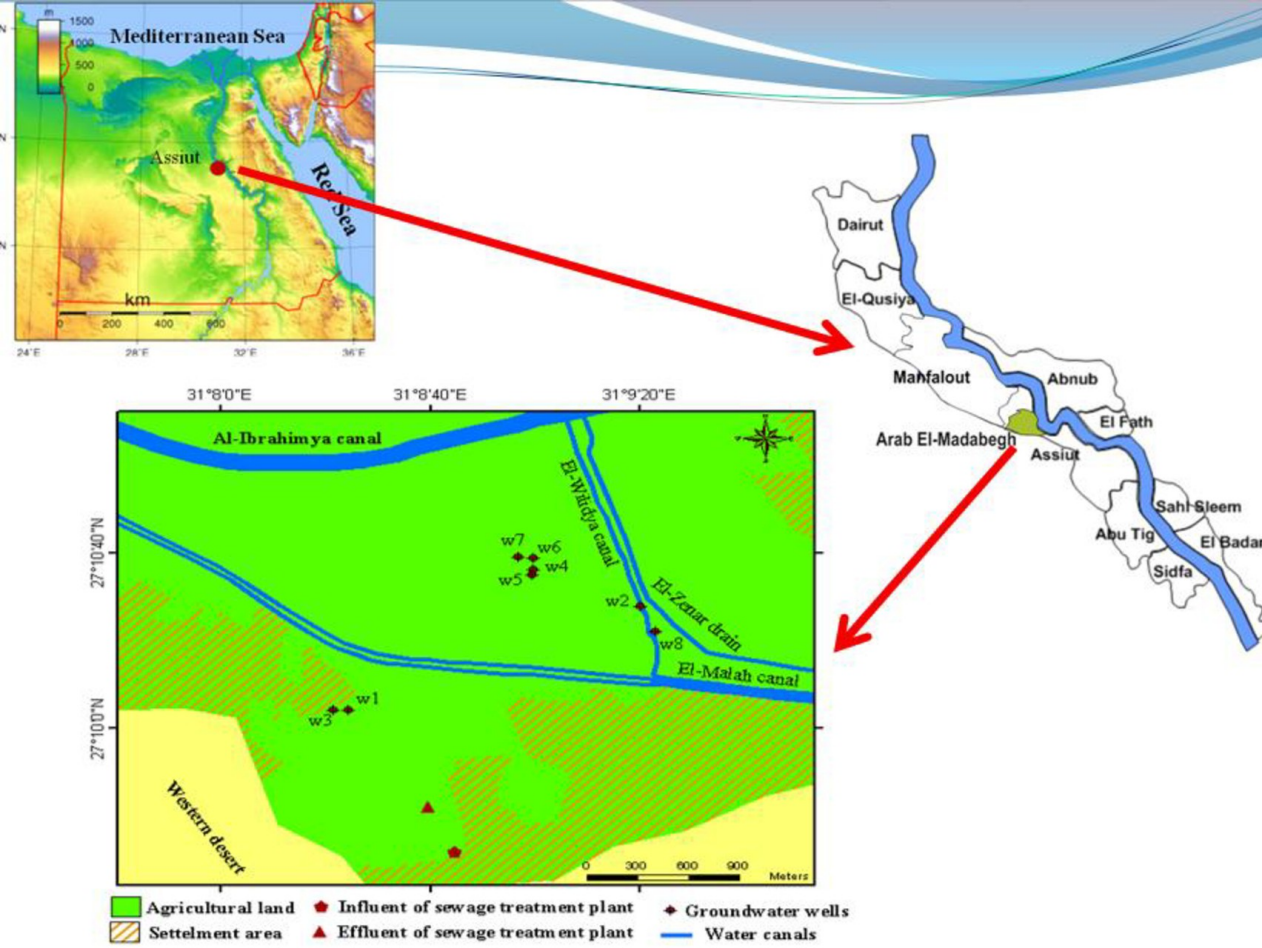
Location map of Arab El-Madabegh area showing its location on a satellite image for Egypt (upper right), its location to Assiut governorate (upper left), and a detailed map for different features (down).

**Fig 4:**
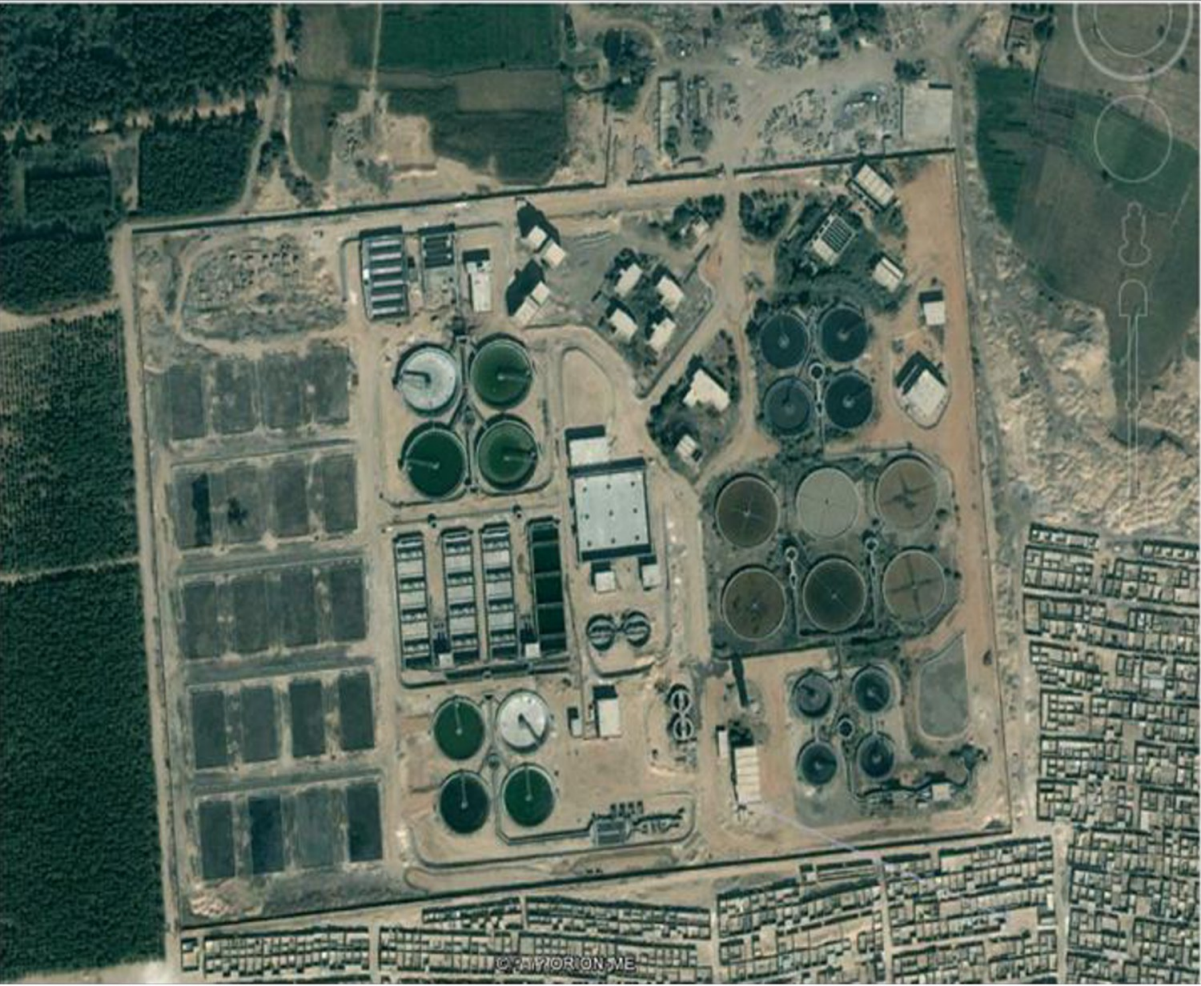
Aerial view of Arab El-Madabegh sewage treatment plant.

The research region is located near the Egyptian city of Assiut, between latitudes 27°09’41.9" and 27°10’39.00", and longitudes 31°08’21.6" and 31°09’23". It is situated northwest of Assiut City as shown in Fig 3. The research region experiences a climate that ranges from semi-arid to arid, with a mean air temperature of around 22° C. During the summer, temperature naturally increases to 30° to 40° C, and fall to less than 13° C during the winter. The amount of rainfall in the studied region is negligible throughout the year. The communities near the Assiut governorate’s scarps suffered significant damage from the wet flash floods and storms that occurred in November 1994. The aerial view of Arab El-Madabegh WWTP shown in Fig 4.

### 2.2 Description of treatment processes

**Fig 5:**
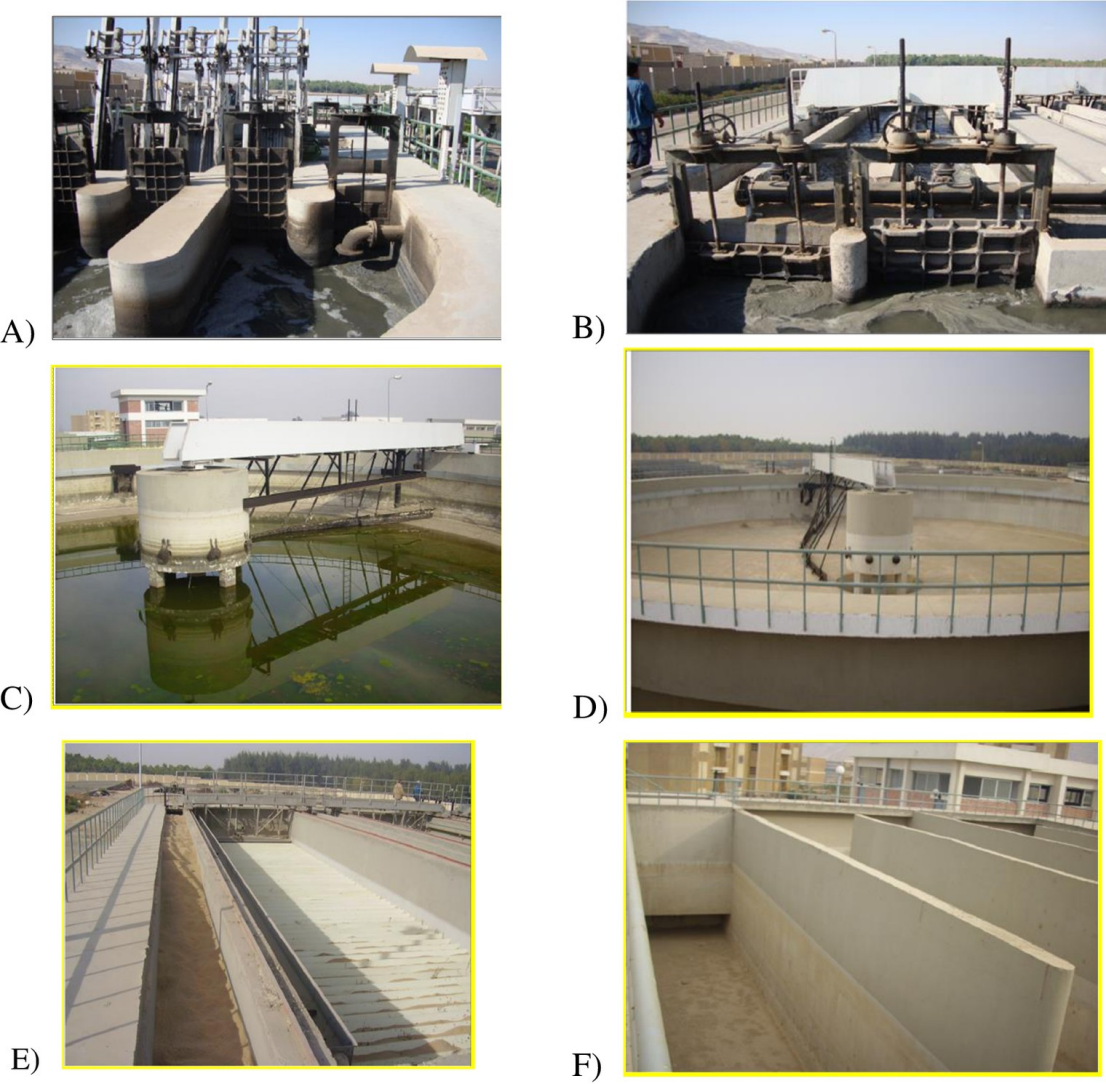
The wastewater treatment processes of Arab El-Madabegh WWTP. A) Bar screens within the treatment plant, B) Grit and oil removal chamber, C) Primary sedimentation tanks, D) Final sedimentation tanks, E) Sand filters, F) Chlorine contact tanks.

The wastewater treatment processes of Arab El-Madabegh WWTP (Fig 5) are explained in stages as the following:

#### 2.2.1 Inlet chamber

It is a rectangular chamber that slows and lowers the pressure and speed of the influent wastewater.

#### 2.2.2 Bar screens

A bar screen is used to filter out any large items carried in the influent wastewater stream, such as plastic packets, rags, cans, sticks, etc. The most popular tool for doing this is an automated mechanically raked bar screen (Fig 5A) or mesh screens with different mesh size to take full advantage of solids removal.

#### 2.2.3 Grit and oil chamber

In the grit and oil removal chamber, (Fig 5B), the influent flow is slowed to allow heavy materials like stones, grit, sand, and gravel to settle to the basin’s bottom and oil and grease to rise to the top to be scrapping off.

#### 2.2.4 Primary settling tanks

During the main sedimentation procedure, wastewater flows through large containers referred to as "primary settling tank” (Fig 5C). These tanks are used to settle sludge. The collected sludge from primary settling tanks is typically continuously driven toward a hopper at the bottom of the tank by mechanically driven scrapers. Primary settling tanks remove around 30% of the dissolved organic matter (BOD) and more than 60% of the suspended solids (TSS) from the wastewater.

#### 2.2.5 Aeration tanks

The aeration tanks provide an ideal environment for aerobic bacteria to digest as much organic debris as possible. The impellers in aeration tanks have two roles: first, circulating air into the tanks for the biological oxidation reactions, and second, mixing air and reactants as needed. Activated sludge from primary sedimentation tanks is also pumped into the aeration tanks. The goal is to remove organic matter with high efficiency, using available oxygen to promote biomass growth. The process can convert ammonia into nitrite, then into nitrate and then into nitrogen gas if all goes well.

#### 2.2.6 Final settling tanks

The final settling tanks, shown in Fig 5D, produce low levels of organic matter and suspended matter as biological floc or filter material settles out, resulting in wastewater water. These tanks are responsible for removing nearly 90% of the suspended organic matter (BOD) and over 95% of the suspended solids (TSS) from the wastewater.

#### 2.2.7 Filtration

Sand and gravel serve as the support media in sand filters (Fig 5E). A sand filtering device is used to remove any remaining suspended particles, turbidity, or organic components.

#### 2.2.8 Disinfection

Chlorine contact tanks (Fig 5F) are used to disinfect the wastewater water from sand filters. Disinfection is employed to reduce the quantity of bacteria in wastewater treatment. The effectiveness of water cleaning is influenced by the method of disinfection used, the concentration and duration of the disinfectant dosage, as well as other environmental factors.

### 2.3 Methodology

The procedures employed to gather and examine water samples from the research region along the sampling methodology, equipment, and the laboratory analysis are described as follows:

#### 2.3.1 Sampling methodology

Twelve treated effluent samples from the Arab El-Madabegh wastewater treatment plant were gathered every two weeks starting in January 2012 and finishing in June 2012 were tested to determine various parameters including pH, Total Dissolved Solids (TDS), Total Suspended Solids (TSS), Temperature (Temp), Conductivity (EC), Turbidity (Turb.), Chemical Oxygen Demand (COD), Biological Oxygen Demand (BOD), Total Organic Carbon (TOC), NO_3_^-^, SO_4_^2-^, Cl^-^, Ca^2+^, PO_4_^3-^, HCO_3_^-^, Na^+^, Mg^2+^, Fe, Mn, K^+^, Cr, Pb, Zn, Ni, Cu, and Cd. The water samples were maintained and kept in a cool container until examination, following standard procedures of the American Public Health Association [39].

#### 2.3.2 Equipment

- The pH, temperature, and conductivity of the field were measured using an ultra-meter (Myron L Company), Model: (6P).
- A total organic carbon analyzer (Shimadzu Corporation, Model: (TOC-V CSN). was used to measure the total organic carbon content of water.
- A UV-Visible spectrophotometer (Shimadzu Corporation), Model: (UV-1650PC) was used to quantify nitrate, nitrite, ammonia, fluoride, phosphate, and sulfate levels.
- A flame spectrophotometer was used to detect salt and potassium concentrations at the Agricultural Faculty laboratory at Assiut University.
- The Shimadzu Corporation (AA-6800) Atomic Absorption Spectrophotometer (AAS) (Flameless type with Flame component) was used to detect Lead (Pb), Cadmium (Cd), Zinc (Zn), Chromium (Cr), Copper (Cu), Manganese (Mn), Iron (Fe), and Nickel (Ni) (GFAAS).

#### 2.3.3 Laboratory analysis techniques

The obtained water samples were chemically tested using American Public Health Association (APHA) recommended analytical procedure. The water quality parameters, units, and analytical methods are provided in Table S1 in S1 File.

### 2.4 Treated wastewater effluents assessment

#### 2.4.1 Permeability Index (PI)

The following formula was used to calculate PI from the average concentrations of HCO_3_^-^, Ca^2+^, Mg^2+^, Na^+^, and K^+^ in the treated wastewater effluent:

$$PI=\frac{({Na}^{+}+K^{+}+\surd HCO3-)*100}{\left({Na}^{+}+K^{+}+{Ca}^{+2}+{Mg}^{+2}\right)}$$ (1)

#### 2.4.2 Soluble Sodium Percentage (SSP)

SSP was calculated using the average amounts of Na^+^, K^+^, Ca^2+^, and Mg^2+^ in treated wastewater effluent using the following formula:

$$SSP=\frac{\left[({\mathrm{Na}}^{+}+K^{+})*100\right]}{\left({\mathrm{Na}}^{+}+K^{+}+{\mathrm{Ca}}^{2+}+{\mathrm{Mg}}^{2+}\right)}$$ (2)

where all ionic concentrations are expressed in meq/L.

#### 2.4.3 Sodium Absorption Ratio (SAR)

SAR was determined using the formula that follows

$$SAR=\frac{{\mathrm{Na}}^{+}}{\sqrt{\frac{{\mathrm{Ca}}^{2+}+{\mathrm{Mg}}^{2+}}{2}}}$$ (3)

It examines the interaction between the soluble forms of sodium, calcium, and magnesium.

#### 2.4.4 Magnesium Hazard (MH)

Ca^2+^, and Mg^2+^ average concentrations from treated wastewater effluent were used to compute MH using the following formula:

$$MH=\frac{{Mg}^{2+}}{{Ca}^{2+}+{Mg}^{2+}}*100$$ (4)

#### 2.4.5 Residual Sodium Carbonate (RSC)

RSC was calculated using the following formula based on the average CO_3_^2-^, HCO_3_^-^, Ca^2+^, and Mg^2+^ contents of treated wastewater effluent:

$$RSC=(C{O_{3}}^{‐2}+HCO^{3‐})–(Ca^{+2}+Mg^{+2})$$ (5)

Where the ions are expressed in meq/l.

## 3. Results and discussions

### 3.1 Characteristics of treated wastewater effluents

The environmental characteristics of the plant’s treated wastewater effluent were measured and compared to the Egyptian permissible limits [40]. The detailed maximum discharge limits for treated wastewater into the aquatic environment can be found in Table S2 in S1 File.

### 3.2 Physiochemical characteristics of treated wastewater effluents

#### 3.2.1 Hydrogen ion concentration (pH)

**Fig 6:**
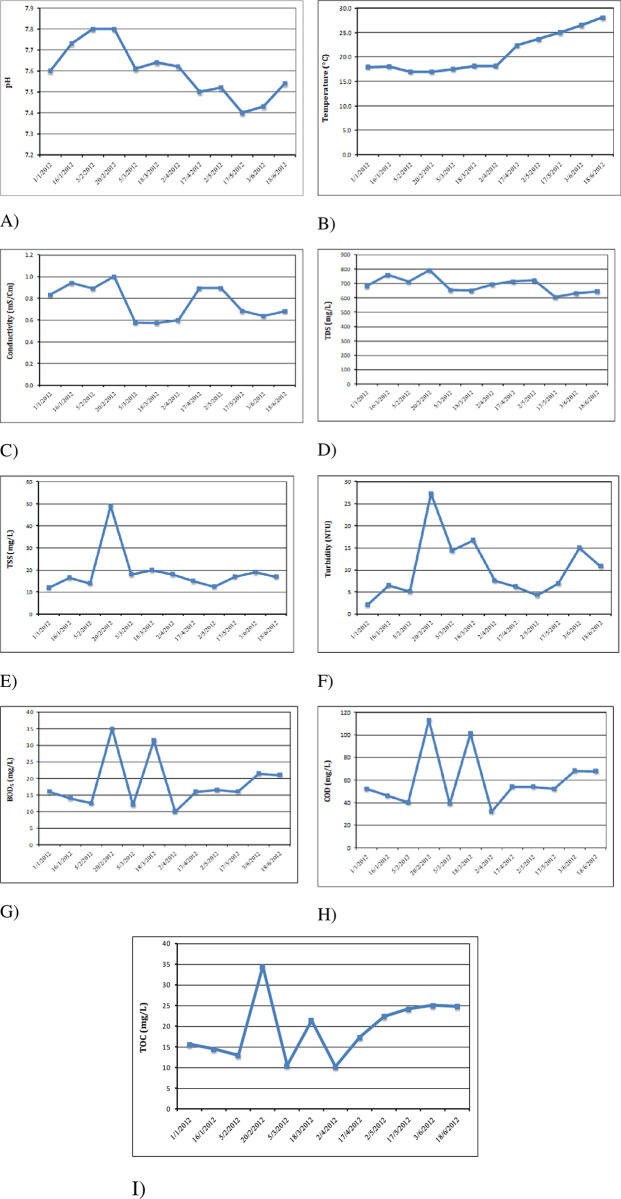
A) Variation in pH values, B) Variation in temperature values, C) Variation in conductivity, D) Variation in total dissolved solids concentrations, E) Variation in total suspended solids concentrations, F) Variation in turbidity concentrations G) Variation in concentrations of biological oxygen demand, H) Variation in concentrations of chemical oxygen demand, I) Variation in concentrations of total organic carbon during the study period.

The Hydrogen ion concentration (pH) levels in the final treated wastewater disposal to the El-Zenar drain ranged from 7.40 to 7.80 (Fig 6A), which was within the permitted range (6–9) for Egyptian standards. A low pH value have an impact on aquatic life and make recreational usage difficult [41]. Numerous other elements are likewise more soluble at low pHs, including Al, B, Cu, Cd, Hg, Mn, and Fe [42]. The toxicity of other pollutants in the river may also be influenced by high pH levels. Additionally, at low pHs, Mn and Fe are more soluble (42). Other pollutants toxicity may be impacted by high pH levels in the river as well. For instance, ammonia is much more toxic in alkaline than in acidic water because free ammonia (NH_3_) at high pH values (pH > 8.5) is more harmful to aquatic biota than oxidized ammonia (NH_4_^+^) and biological treatment units are also affected by damage from severe pH [43].

#### 3.2.2 Temperatures

As shown in (Fig 6B), the average temperature in the final treated wastewater is 20.8 ˚C, ranging between 17 ˚C and 28 ˚C. The average temperature was 35 degrees which is lower than Egypt’s permitted limit for dumping in the Al-Zinar Canal. Temperature determines the solubility and consequently the availability of oxygen in water, so any rise in the average temperature of a body of water can have an ecological impact [44].

#### 3.2.3 Electrical conductivity (EC)

In Fig 6C, the electrical conductivity (EC) ranges between 0.57 mS/cm and 1.00 mS/cm in the final treated wastewater that was released without the necessary treatment, resulting in the highest possible rating due to the power failure on February 20th. Salts from domestic wastewater are regularly dissolved in wastewater effluents. The salinity of wastewater effluents can increase due to high salt concentrations in wastewater effluents, which can harm freshwater aquatic ecosystems [45, 46].

#### 3.2.4 Total Dissolved Solids (TDS) and Total Suspended Solids (TSS)

The agricultural water quality parameter TDS is crucial for determining the salinity of the soil [47]. The average TDS concentration is 688 mg/L, with varied values ranging from 605 mg/L to 792 mg/L (Fig 6D). Consequently, the final effluent concentrations are within the appropriate range of the WHO standards and Egypt’s (2000 mg/L) TDS limits [44]. Both TDS and EC serve as indicators of salinity in effluents, and the TDS levels in the final effluent during this study were lower than those reported by Odjadjare and Okoh [47], but higher than those reported by Osode and Okoh [48]. Elevated TDS concentrations reduce photosynthesis, raise water temperatures, impact water clarity, killing aquatic organisms by causing osmotic shock and impairing their ability to control their osmotic pressure [47].

Another crucial factor in controlling wastewater discharge is TSS. TSS concentrations ranged between 12 mg/L and 49 mg/L during the study period (Fig 6 E), with an average TSS is 19 mg/L. As a result, the final effluent concentrations are acceptable compared to the Egyptian limit of 50 mg/L for TSS. This value is comes from the study of El-Gohary et al. [49], in which the mean residual TSS was 21 mg/L and the corresponding percentage removal value was 85%. Maximum values were observed on February 20th due to a power outage on that day. TSS impacts irrigation systems negatively by clogging pipes, sprinklers, emitters, and restricted water pathways. TSS also contribute to heavy metal adsorption, creation of formations containing heavy metals [50]. In water bodies with high TSS, sunlight intensity is reduced, affecting primary productivity particularly for green algae, and disturbing the balance of the aquatic food chain. Temperature in the aquatic environment is also influenced by light levels, harming the primary and secondary productivity of aquatic life. So, the system’s capacity can’t keep the temperature steady. According to Nkegbe et al. [51], TSS is a source of odor brought on by organic degradation, leading to sludge accumulation and anaerobic conditions in the water supply [52].

#### 3.2.5 Turbidity (Turb.)

The final treated wastewater’s turbidity ranged between 2.14 NTU and 27.30 NTU, and the average turbidity is 10.27 NTU (Fig 6F). Turbidity is correlated with TSS and total coliform reduction [53]. Sand filters and sedimentation tanks both contribute to reducing turbidity, in addition to TSS. The maximum value was observed on February 20th since that day the electricity was shut off.

#### 3.2.6 Organic content (COD, BOD_5_, and TOC)

Organic matter can be characterized using three parameters: Biological Oxygen Demand (BOD), Chemical Oxygen Demand (COD), and Total Organic Carbon (TOC). BOD and COD, essential biochemical parameters, are commonly employed to evaluate wastewater quality, which reflect its organic load [15]. COD employ potent oxidizing agents to measure pollutant loading in order to effectively identify chemical-oxygen-demanding qualities in watersheds [54]. Unlike BOD and COD, total organic carbon (TOC) gauges the oxygen requirement for organic matter decomposition [55]. Effluents with high organic content, particularly BOD, can deplete natural dissolved oxygen in aquatic habitats. The significant drop in organic matter was caused by sand filters, aeration tanks, and sedimentation tanks throughout the research period, with concentrations varied from 10.0 mg/L to 35.0 mg/L for BOD_5_, 10.2 to 34.5 mg/L for TOC, and 32 mg/L to 113 mg/L for COD (Fig 6I). The average levels of COD and BOD_5_ in the treated effluent complied within the recommended flow limits of 60 mg/L for COD and 80 mg/L for BOD_5_ according to the Egyptian guidelines (Fig 6G, 6H). The highest levels of organic matter were observed on February 20^th^ due to switching off the power.

#### 3.2.7 Nutrients (PO_4_^3-^, and NO^3-^)

**Fig 7:**
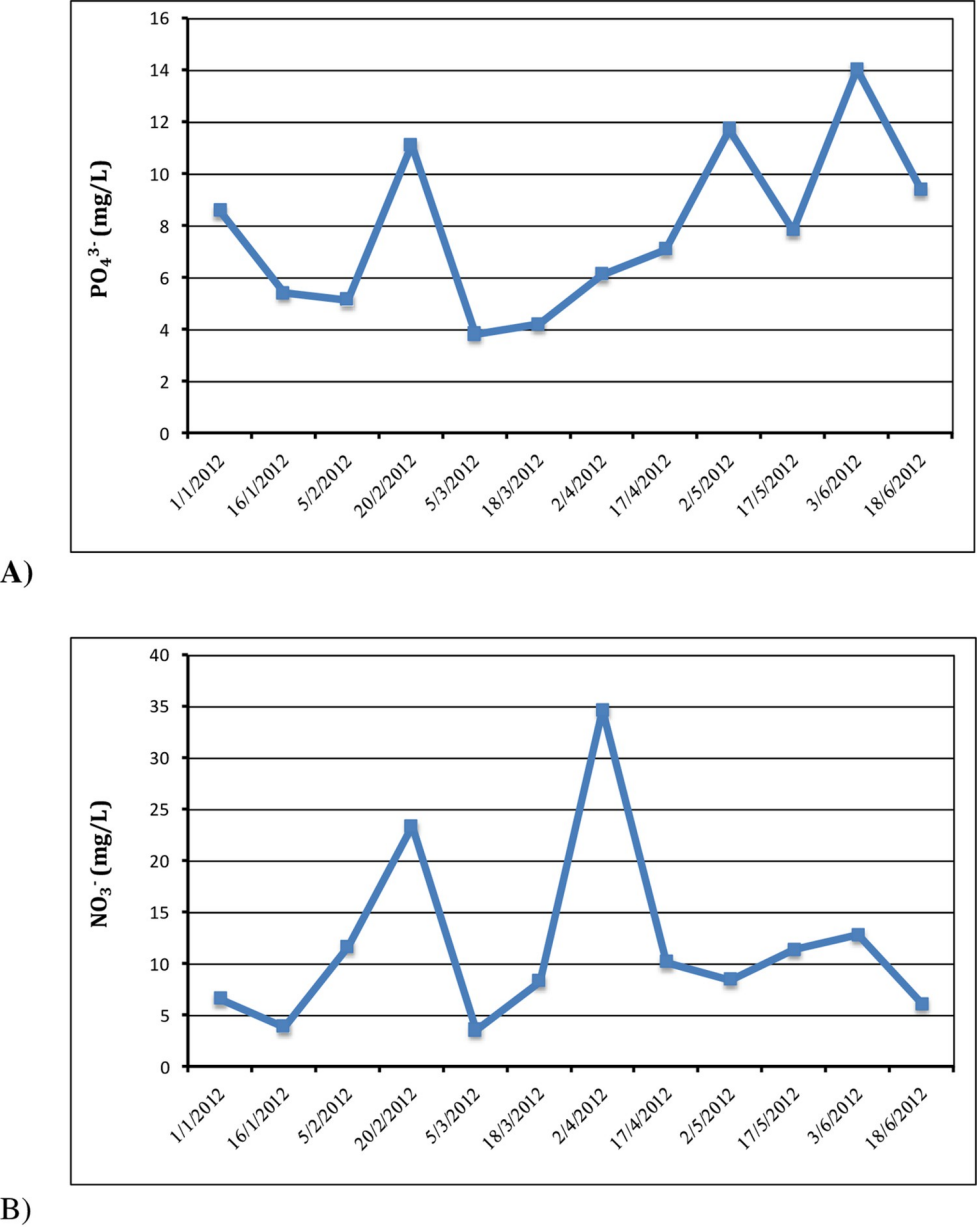
Variation in nutrients. (A) phosphate and (B) nitrate concentrations during the study period.

The biological treatment process employs microorganisms to absorb nutrients. Fig 7A depicts the range of phosphorus concentrations over the research period, which varying between (3.8 mg/L and 14.1 mg/L), with an average concentration of 7.9 mg/L. The average phosphate content exceeded Egypt’s permitted 2 mg/L limit. According to Narr et al. [56], no persistent patterns in nutrients concentrations were observed across test days. While there are no suggested limitations for nitrate, exceeding permissible limit of 10 mg/L may be harmful due to its impact on the environment and human health [57, 58];. The average nitrate concentration through of the study was 11.8 mg/L, with values between 3.6 mg/L and 34.7 mg/L (Fig 7B). Phosphorus is the only plant nutrient that, when supplied to an aqueous environment, may promote plant growth. The high nitrate content causes eutrophication when phosphates are present in the water at a concentration of 0.1 mg/L, [51, 59]. controlling phosphorus discharge from municipal and commercial wastewater treatment facilities is crucial to prevent the surface waters eutrophication [60].

#### 3.2.8 Major anions

*3*.*2*.*8*.*1 Chloride (Cl-) and Sulfate (SO*_*4*_^*2-*^*)*. The treated wastewater exhibited an average chloride content of 75.4 mg/L, ranging from 62.9 mg/L ~ 83.9 mg/L. The high chloride concentration adversely affected the aquatic species in freshwater. Therefore, osmoregulation is a biological mechanism used by aquatic species to maintain the appropriate level of salt and other solutes, becomes compromised, impacting survival, reproduction, and growth [61].

No specific standards exist for chloride and sulfate in discharged effluent. The average Sulfate level was 83 mg/L, ranging from 39 mg/L ~ 105 mg/L. Increased sulfate concentrations are known to be toxic to aquatic life in freshwater environments. Sulfate concentrations above a certain point are toxic to aquatic communities, potentially disrupting osmoregulation and harming aquatic communities.

*3*.*2*.*8*.*2 Bicarbonate (HCO*_*3*_^*-*^*) and Carbonate (CO*_*3*_^*2-*^*)*. Concentrations of bicarbonate and carbonate serve as indicators of alkalinity. industrial and municipal wastewater must undergo treatment before flowing into lakes, canals, and rivers, as high alkalinity levels can result in substantial sludge production. Alkalinity representing a water body’s buffering capacity [62], can be influenced by the addition of cleaning solutions and soap-based products to domestic water supplies, leading to increased alkalinity and bicarbonate levels. This can increase the alkalinity and bicarbonate levels of the water by increasing the alkalinity. Biological nitrification in aeration tanks and chlorination for effluent disinfection can consume bicarbonate during treatment processes.

**Fig 8:**
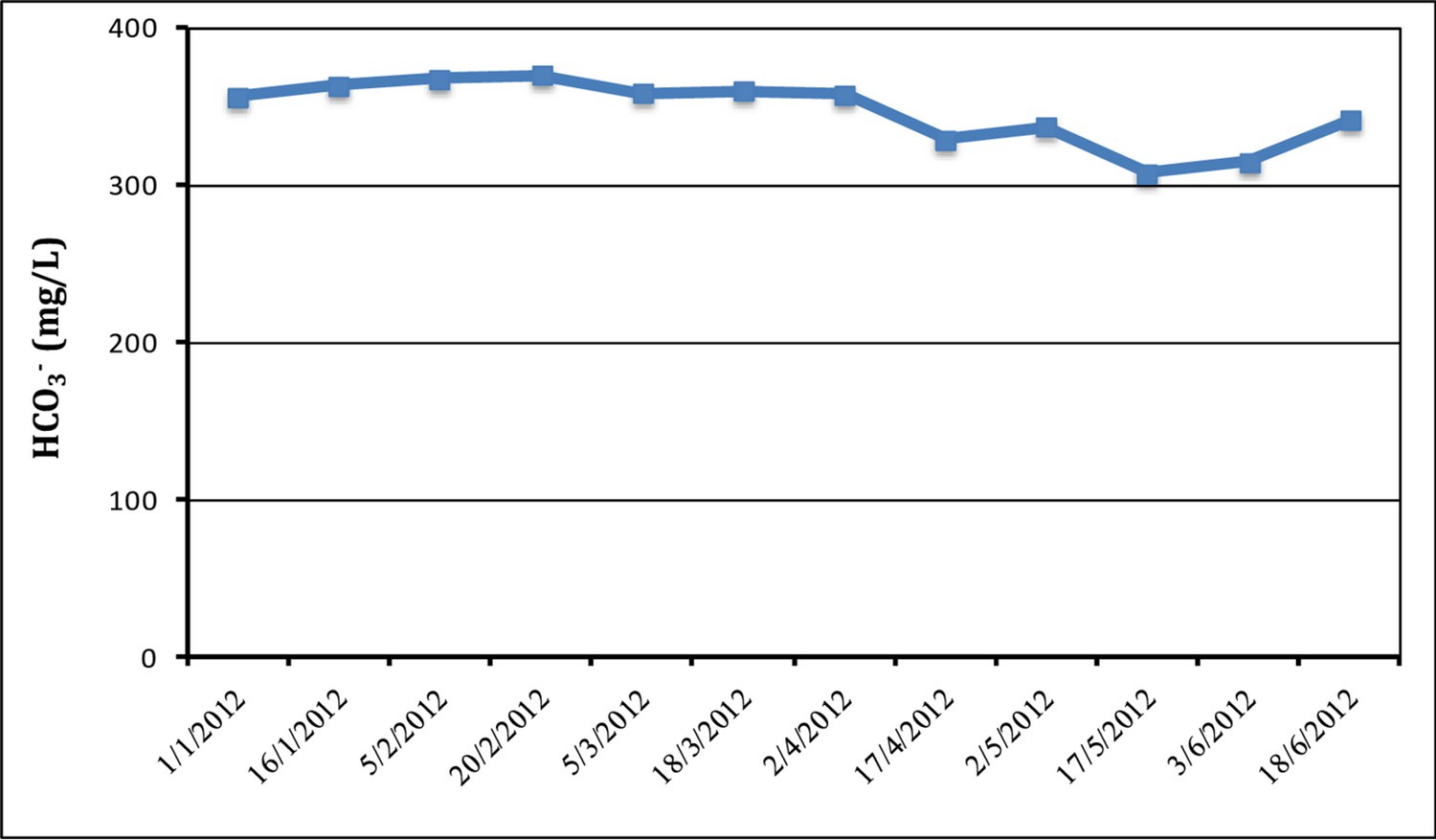
Variation in bicarbonate concentrations during the study period.

As revealed in Fig 8, the average bicarbonate content in the effluent diversed between 307.4 mg/L and 369.7 mg/L, while carbonate concentrations were non-negligible throughout the research duration. There are no specified limitations for bicarbonate content in wastewater discharge. Table 1 summarizes the chemical analysis results from the Arab El-Madabegh WWTP.

**Table 1:** The chemical analyses of Arab El-Madabegh WWTP.

Date	pH	Temp (°C)	Conductivity (mS/cm)	TDS (mg/L)	Turbidity (NTU)	TSS (mg/L)	COD (mg/L)	BOD_5_(mg/L)	TOC (mg/L)	NO_3_^-^ (mg/L)	PO_4_^3-^ (mg/L)	Alkalinity (mg Caco_3_/L)	HCO_3_^-^ (mg/L)	CO_3_^-^ (mg/L)	SO_4_^2-^(mg/L)	Cl^-^ (mg/L)	F^-^(mg/L)
01/01/2012	7.6	18	0.832	680.98	2.14	12	52	16	15.6	6.6	8.6	292	356.2	0	99	77	0.52
16/01/2012	7.73	18.1	0.941	759.42	6.54	16.5	46	14	14.5	3.9	5.4	298	363.6	0	82	82.9	0.49
05/02/2012	7.8	17	0.89	710.93	5.14	14	40	12.5	13	11.7	5.1	301	367.8	0	96	83.9	0.58
20/02/2012	7.8	17	1	792	27.3	49	113	35	34.5	23.4	11.1	303	369.7	0	39	77.5	0.7
05/03/2012	7.61	17.5	0.578	655	14.4	18	39	12	10.5	3.6	3.8	294	358.7	0	85	75.3	0.41
18/03/2012	7.64	18.2	0.574	650	16.7	20	101	31.5	21.4	8.3	4.2	295	359.9	0	72	77.8	0.48
02/04/2012	7.62	18.2	0.598	693	7.62	18	32	10	10.2	34.7	6.1	293	357.5	0	87	80.8	0.43
17/04/2012	7.5	22.4	0.892	713	7.62	15	54	16	17.3	10.2	7.1	270	329.4	0	80	62.9	0.54
02/05/2012	7.52	23.7	0.894	720	6.26	12.5	54	16.5	22.4	8.5	11.7	276	336.7	0	105	82.3	0.52
17/05/2012	7.4	25	0.683	605.07	4.29	17	52	16	24.2	11.4	7.8	252	307.4	0	72	69.8	0.54
03/06/2012	7.43	26.5	0.637	631.77	7	19	68	21.5	25	12.8	14.1	258	314.8	0	90	68.8	0.48
18/06/2012	7.54	28.1	0.682	644.43	15	17	67.7	21	24.8	6.1	9.4	280	341.6	0	89	65.8	0.51
**Min**	7.4	17	0.574	605.07	2.14	12	32	10	10.2	3.6	3.8	252	307.4	0	39	62.9	0.41
**Max**	7.8	28.1	1	792	27.3	49	113	35	34.5	34.7	14.1	303	369.7	0	105	83.9	0.7
**Average**	7.6	20.8	0.8	688.0	9.4	18.5	59.9	18.5	19.5	11.8	7.9	284.3	346.9	0.0	83.0	75.4	0.52

#### 3.2.9 Correlation between physiochemical variables of treated wastewater effluents

The relationships between different treated wastewater effluents were investigated using Pearson correlation analysis (Table 2). With correlation values ranging from 0.72 to 0.93, 0.94, and 0.67, the results illustrated strong positive relationships between pH and the following assessed treated wastewater quality ions: TDS, Alkalinity, Sulfates, and Chloride. Nonetheless, there are notable inverse relationships between temperature and pH. These important correlations demonstrate the role of these ions in the mineralization of treated wastewater effluents. Moreover, there was a strong correlation between each of the previously listed ions related to treated wastewater effluents. Furthermore, turbidity has an inverse relationship with organic variables such as TSS, COD, BOD_5_, and TOC, with correlation values that fluctuate from 0.85 to 0.77, 0.57, and 0.55 [32]. However, there are significant negative relationships between turbidity and sulfate. Temperature has a negative relationship with TDS, Alkalinity, Sulphate, and Chloride, with correlation values ranging from r = 0.54,0.84, 0.84, and 0.68. Temperature and PO_4_^3-^ have a high relationship (r = 0.6). The significant positive relationship between conductivity and TDS (r = 0.82) demonstrated that treated wastewater effluents are saline in nature [63]. TDS has a positive relationship with alkalinity, sulfate, and iron (r = 0.62, 0.62, and 0.54, respectively). Organic matter (BOD_5_, TOC) has a significant relationship with COD. BOD_5_ has a strong relationship (r = 0.82). Also TOC and PO_4_^3-^ has a strong relationship (r = 0.69), which suggested that treated wastewater effluents used can improve the soil fertility of semiarid area due to greater phosphate levels [64]. Treated wastewater effluents is characterized by relatively high levels of biodegradable organic matter which can be used to improve soil fertility [65, 66].

**Table 2:** Correlation matrix for the chemical analyses of Arab El-Madabegh WWTP.

Variables	pH	Temp (°C)	Conductivity y (mS/cm)	TDS (mg/L)	Turbidity (NTU)	TSS (mg/L)	COD (mg/L)	BOD_5_ (mg/L)	TOC (mg/L)	NO_3_^-^ (mg/L)	PO_4_^3-^ (mg/L)	Alkalinity (mg Caco_3_/L)	HCO_3_^-^ (mg/L)	SO_4_^2-^ ^-^(mg/L)	Cl- (mg/L)	F- (mg/L)
pH	1															
Temp (0C)	-0.802	1														
Conductivity	0.455	-0.226	1													
TDS	0.723	-0.538	0.822	1												
Turbidity	0.411	-0.192	0.022	0.313	1											
TSS	0.436	-0.259	0.278	0.480	0.849	1										
COD	0.184	0.010	0.123	0.179	0.768	0.733	1									
BOD_5_	0.185	0.012	0.103	0.164	0.767	0.732	0.999	1								
TOC	-0.131	0.420	0.243	0.085	0.554	0.661	0.819	0.818	1							
NO_3_^-^	0.170	-0.202	-0.059	0.238	0.205	0.445	0.064	0.072	0.079	1						
PO4_3_^-^	-0.392	0.600	0.228	0.025	0.043	0.258	0.326	0.330	0.692	0.143	1					
Alkalinity	0.934	-0.838	0.259	0.617	0.403	0.309	0.119	0.120	-0.286	0.126	-0.492	1				
HCO_3_^-^	0.935	-0.838	0.260	0.617	0.401	0.308	0.116	0.118	-0.287	0.126	-0.493	1.000	1			
SO_4_^2-^	-0.305	0.240	-0.164	-0.313	-0.770	-0.891	-0.702	-0.695	-0.583	-0.337	0.002	-0.173	-0.171	1		
Cl-	0.676	-0.687	0.253	0.485	-0.060	0.031	-0.145	-0.136	-0.320	0.174	-0.287	0.674	0.675	0.135	1	
F-	0.382	-0.094	0.740	0.542	0.395	0.654	0.560	0.549	0.674	0.175	0.364	0.132	0.134	-0.563	0.016	1

### 3.3 Heavy and trace metals

The importance of heavy and trace metals in water cannot be overstated. These metals (Ca, Co, Cr, Cu, Fe, K, Mg, Mn, Na, Ni, and Zn) are needed by living organisms in varying amounts as macro- or micronutrients for sustained growth [67]. Table 3 displays analytical results of Ca, Mg, Na, K, Fe, Mn, Pb, Cd, Zn, Cu, Ni, and Cr, selected as representative trace metals. The concentration of these metals in wastewater is influenced by various regional factors, including the observed industries, social standards, and environmental awareness regarding the effects of inadequate waste disposal [18, 68, 69].

**Table 3:** Results of heavy and trace metals of Arab El-Madabegh WWTP.

Heavy metals	Ca ^+2^ (mg/L)	Mg ^2+^ (mg/L)	Na + (mg/L)	K + (mg/L)	Fe (mg/L)	Mn (mg/L)	Pb (mg/L)	Cd (mg/L)	Zn (mg/L)	Cu (mg/L)	Ni (mg/L)	Cr (mg/L)
01/01/2012	37.4	13.1	130	23	0.0009	0.023	0.0014	0.00004	0.0004	0.00377	0.005	0.00017
16/01/2012	38	13.4	131.5	22	0.0098	0.021	0.0012	0.000004	0.0011	0.00045	0.029	0.00142
05/02/2012	39	13.3	133	25	0.0103	0.031	0.0029	0.000006	0.0008	0.0061	0.008	0.00014
20/02/2012	38	13.2	135	26	0.0006	0.026	0.0025	0.000043	0.0007	0.04221	0.019	0.00052
05/03/2012	36.4	12.7	129	19	0.0117	0.031	0.003	0.000012	0.0002	0.0012	0.01	0.00201
18/03/2012	34.4	12	129	22	0.0113	0.031	0.0029	0.000003	0.0007	0.00002	0.008	0.00075
02/04/2012	36.8	13	130	23	0.0096	0.022	0.0029	0.000006	0.0006	0.00009	0.009	0.00127
17/04/2012	36	13.4	128	19	0.0137	0.028	0.0023	0.000014	0.0006	0.00017	0.011	0.00082
02/05/2012	36	12.6	130.5	23.5	0.0083	0.028	0.0016	0.000003	0.0005	0.00006	0.011	0.00119
17/05/2012	34.7	12.2	129.99	20.2	0.0158	0.03	0.0023	0.000017	0.0009	0.00102	0.016	0.00003
03/06/2012	41.6	11.5	127.2	19.5	0.011	0.03	0.002	0.000002	0.0007	0.00034	0.001	0.00186
18/06/2012	43.8	12.1	124	18	0.0102	0.029	0.0021	0.000007	0.0001	0.00015	0.016	0.00073
**Min**	34.4	11.5	124	18	0.0006	0.021	0.0012	0.000002	0.0001	0.00002	0.001	0.00003
**Max**	43.8	13.4	135	26	0.0158	0.031	0.003	0.000043	0.0011	0.04221	0.029	0.00201
**Average**	37.7	12.7	129.8	21.7	0.0094	0.028	0.0	0.000013	0.000608	0.004632	0.012	0.00091

The average levels of heavy metals in the effluent treated wastewater were below the permissible limits, with the exception of lead was lower than the Egyptian suggested effluent levels in Table 2. Lead concentrations varied from 0.0012 mg/L to 0.0030 mg/L. Cadmium levels ranged from 0.000002 mg/L to 0.000043 mg/L and Zinc values ranged from 0.0001 mg/L to 0.0011 mg/L while Copper values from 0.00002 mg/L to 0.04221 mg/L during the research period. The amounts of nickel varied from 0.001 mg/L to 0.029 mg/L, and the values of chromium varied from 0.00003 mg/L to 0.00201 mg/L. This result contradict from [22] who demonstrated that the levels of Zn, Pb, Cd, and Ni in tested plants’ edible sections of lettuce and spinach exceeded permissible limit. However, the levels of Cu were within the safe limit.

The concentrations of iron, potassium, salt, calcium, manganese, and magnesium in effluent discharges are not restricted. The range of iron concentrations was between 0.0006 and 0.0158 mg/L, with a mean of 0.0094 mg/L. The average manganese concentration was 0.028 mg/L, with values ranging between 0.021 mg/L and 0.031 mg/L. The average sodium content was 129.77 mg/L, with the range between 124.00 mg/L and 135.00 mg/L. Potassium concentrations were between 18 and 26 mg/L, a mean of 21.68 mg/L. The average calcium level was 37.68 mg/L, and varying between 34.40 mg/L and 43.80 mg/L. Magnesium concentrations were between 11.52 and 13.44 mg/L, a mean of 12.71 mg/L.

Using Pearson correlation analysis (Table 4), the associations between various heavy metals were examined (Table 4). The findings showed a substantial positive correlation between mg and the trace elements (Na and K) in treated wastewater effluents (with r = 0.6, 0.49 respectively). In the other hand, a weak positive correlation between mg and the trace elements (Cd, Cu and Ni) in treated wastewater effluents (with r = 0.36, 0.31, and 0.4 respectively). However, Mg and Mn have some noteworthy negative interactions. Moreover, Na and K have a good relationship. Furthermore, The results showed a substantial positive correlation between Na and the trace elements (K, Zn and Cu) in treated wastewater effluents (with r = 0.88, 0.56, 0.65 respectively). In the other hand, a weak positive correlation between Na and the trace elements (Cd and Ni) in treated wastewater effluents (with r = 0.43 and 0.28 respectively). Inversely, Na and K have agood negative correlation. Additionally, K and Cu have a strong positive correlation and astrong negative correlation with Fe. However, Fe has a negative relationship with CD, Cu. In the other hand Fe has aweak positive correlation with Mn and Pb (r = 0.48 and 0.29 respectively).

**Table 4:** Correlation matrix for heavy and trace metals of Arab El-Madabegh WWTP.

Variables	Ca ^+2^ (mg/L)	Mg ^2+^ (mg/L)	Na ^+^ (mg/L)	K ^+^ (mg/L)	Fe (mg/L)	Mn (mg/L)	Pb (mg/L)	Cd (mg/L)	Zn (mg/L)	Cu (mg/L)	Ni (mg/L)	Cr (mg/L)
Ca^2+^(mg/L)	1											
Mg^2+^(mg/L)	-0.230	1										
Na^+^(mg/L)	-0.411	0.603	1									
K (mg/L)	-0.241	0.488	0.880	1								
Fe (mg/L)	-0.159	-0.346	-0.457	-0.639	1							
Mn(mg/L)	0.019	-0.541	-0.272	-0.327	0.488	1						
Pb (mg/L)	-0.207	-0.088	0.075	0.031	0.293	0.515	1					
Cd (mg/L)	-0.109	0.361	0.431	0.376	-0.727	-0.289	-0.124	1				
Zn (mg/L)	-0.318	0.214	0.561	0.392	0.162	-0.234	-0.160	-0.110	1			
Cu (mg/L)	0.050	0.306	0.652	0.599	-0.655	-0.113	0.138	0.708	0.114	1		
Ni (mg/L)	-0.020	0.412	0.279	0.085	-0.010	-0.410	-0.318	0.072	0.372	0.265	1	
Cr (mg/L)	0.149	-0.221	-0.283	-0.378	0.203	-0.030	0.002	-0.480	-0.174	-0.266	-0.063	1

### 3.4 Evaluation of treated wastewater effluent for irrigation

Arab El-Madabegh treated effluent was determined to be appropriate for irrigation system in Egypt [40] and Food and Agricultural Organization [70] regulations for unrestricted irrigation.

The average pH level was 7.6 mg/L, within FAO regulations range of 6.5–8 mg/L. According to FAO regulations, the average TDS concentration was 688.02 mg/L, less than 2000 mg/L permissible level in Egypt, characterized as having a low to moderate degree of limitation for reuse in irrigation. There was an average BOD5 concentration of 18.5 mg/L in the tertiary-treated wastewater, which is below the 20 mg/L permissible level in Egypt. Nonetheless, the COD content (59.9 mg/L) exceeded the Egyptian-permitted limit of 40 mg/L. Proper operation of the sand filtering and aeration stages in the plant may further decreased the COD values. This discrepancy may be the result of operational concerns. TSS had an average value of 19 mg/L which is lower than the 20 mg/L permissible level in Egypt.

The average Cl- concentration was 75.4 mg/L, below Egypt’s 300 mg/L maximum allowable standard. The average NO_3_^-^ (as N) level in the effluent was 2.7 mg/L, below the FAO guideline of 5 mg/L. Moreover, average F^-^ levels were 0.52 mg/L, below the FAO limit of 1 mg/L. Furthermore, the average Fe concentrations were 0.0094 mg/L, below the FAO guideline of 5 mg/L. The average Mn content was 0.028 mg/L, below the FAO guideline of 0.2 mg/L.

Heavy metal concentrations such as Cd, Cr, Cu, Ni, Pb, and Zn averaged 0.000013 mg/L, 0.00104 mg/L, 0.00463 mg/L, 0.012 mg/L, 0.0023 mg/L, and 0.0006 mg/L, respectively, satisfying Egyptian and FAO standards of 0.01 mg/L, 0.1 mg/L, 0.2 mg/L, 0.2 mg/L, 5 mg/L, and 2 mg/L. These results were in agreement with Sherif [71], where concentrations of macro, micro, and heavy metals examined in water samples from canal Bani Ghalib in Assiut Governorate, Egypt, were within acceptable levels. While Cd, Co, and Cr were below acceptable levels, and available B and Ni were at moderate amounts, the available Fe, Mn, Zn, and Cu Pb in soils exceeded legal limits. However, Roshdy [72] proved that heavy metal contamination existed in the soil of the communities of Ellwan, Mangabad and El-Madabegh villages in Assiut Governorate. The results obtained revealed that the concentrations of Pb, Cd, and Ni in the edible plants were greater than the permissible limit levels, while those of Zn and Cu were within the safe limit values. Table 5 lists the Maximum allowable standards for water quality for Irrigation.

**Table 5:** Maximum guidelines permitted of treated wastewater effluent for irrigation.

Parameter	Unit	Tertiary treated effluent*	Egyptian limit (unrestricted crop)	FAO limit
				Degree of restriction on use
				None Slight to Sever moderate
**pH**	unit	7.6	-	6.5–8
**COD**	mg/L	59.9	40	-
**TSS**	mg/L	19	20	-
**BOD5**	mg/L	18.5	20	-
**Cl-**	mg/L	75.4	300	-
**TDS**	mg/L	688.02	2000	< 450 450–2000 > 2000
**NO**_**3**_^**—**^ **N**	mg/L	2.7	-	< 5 5–30 > 30
**F** ^**-**^	mg/L	0.52	-	1
**Cd**	mg/L	0.000013	0.01	0.01
**Cr**	mg/L	0.00104	0.1	0.1
**Cu**	mg/L	0.00463	0.2	0.2
**Ni**	mg/L	0.012	0.2	0.2
**Pb**	mg/L	0.0023	5	5
**Zn**	mg/L	0.0006	2	2
**Fe**	mg/L	0.0094	-	5
**Mn**	mg/L	0.028	-	0.2

* Each value represents an average of 12 samples taken during 2012

In order to classify treated wastewater quality and assess its suitability for irrigation, the salinity index (SI), permeability index (PI), soluble sodium percentage (SSP), sodium absorption ratio (SAR), magnesium ratio (MR), magnesium hazard (MH), and residual sodium carbonate (RSC) were calculated and classified for average concentrations of treated wastewater effluent in accordance with Table 6

**Table 6:** Calculated irrigation quality parameters of tertiary treated effluent.

Parameter	Value*
EC (μS/cm)	767
Na^+^ (meq/l)	5.64
K^+^ (meq/l)	0.55
Mg^2+^ (meq/l)	1.05
Ca^2+^ (meq/l)	1.88
CO_3_^2-^ (meq/l)	0
HCO_3_^-^ (meq/l)	5.69
PI (%)	67.9
SSP (%)	67.9
SAR	4.66
MR	0.56
MH	35.7
RSC	2.76

* Each value reflects the average of 12 samples collected in 2012.

#### 3.4.1 Salinity Index (SI)

The rise in soil salinity can lead to a long-term decline in productivity, which is the most significant adverse effect wastewater has on the ecosystem. It is evident that irrigation with salty water can increase the salt concentration in the soils, and that this concentration rise may cause an issue that is destructive to the crop or the landscape. The use of wastewater must, therefore, be combined with salinization-control strategies like soil washing and proper soil drainage. Hence, class II irrigation water is defined as wastewater with a medium salinity when utilized as irrigation water, with an average EC value of 767 S/cm. According to Mills [74]; Singh et al. [75] the Classification of irrigation water based on EC values is shown in Table S3 in S1 File.

#### 3.4.2 Permeability Index (PI)

The calculated PI value (67.9%) indicates that the value falls into class II (25% - 75%). Class II suggests the treated wastewater is suitable for irrigation.

#### 3.4.3 Soluble Sodium Percentage (SSP)

According to SSP calculations, irrigation with treated wastewater is dubious in terms of SSP, yielding a score of 67.9%. The water Quality classification based on SSP according to Todd and Mays [73] is shown in Table S4 in S1 File.

#### 3.4.4 Sodium Absorption Ratio (SAR)

SAR was determined to be 4.66, showing that it belongs to class II, indicating low sodium for irrigation use. According to Mills [74]; Singh et al. [75], the classification of irrigation water based on SAR values is shown in Table S5 in S1 File

#### 3.4.5 Magnesium ratio (MR)

Based on the residual Mg/Ca ratio, the MR value was determined to be 0.56, indicating that treated wastewater is suitable for use in irrigation (MR <1.5). According to Paliwal [76], The limits of residual Mg/Ca ratio in irrigation water are shown in Table S6 in S1 File.

#### 3.4.6 Magnesium Hazard (MH)

The calculated MH value (35.7%) is less than 50%. SO, the treated wastewater can be categorized as appropriate for irrigation use in terms of MH.

#### 3.4.7 Residual Sodium Carbonate (RSC)

The treated wastewater is not acceptable for irrigation purposes because the computed RSC value (2.76) is greater than 2.5. According to [77], the classification of irrigation water based on RSC values is shown in Table S7 in S1 File.

## 4. Conclusion and recommendations

The three wastewater treatment facilities are the main source of concern due to the incapability of two of them to perform the secondary treatment. As a result, all influent wastewater from these plants is directed to the tertiary plant, which then releases raw effluent onto nearby agricultural areas. Increased levels of lead and phosphate were observed, posing a contamination threat to the aquatic water body. Elevated lead concentrations can have negative effects on the environment and human health. as well, high phosphate levels contribute to nutrient enrichment. Given this, utilizing treated wastewater in agriculture in an environmentally friendly manner may preserve the quality of the receiving water body while minimizing detrimental effects. Fluctuation in treated effluent concentrations in various parameters throughout the length of the study period indicate that, in some instances, discharge occurs without the recommended treatment, likely due to intermittent electricity supply. It is clear from the variance in parameter concentrations during the duration of the investigation that the plant’s poor performance is attributed to operational, managerial, and design issues. Analyzing the suitability of Arab El-Madabegh treatment plant’s tertiary treated wastewater effluent for irrigation reveals its compliance with the majority of physiochemical requirements. The final tertiary treated effluent continuously complies with the rules given by the Egyptian and FAO for unrestricted irrigation, Despite the average value of COD content in the effluent exceeding the Egyptian allowed limit. This could be the result of certain operational issues. Improved efficiency in the plant’s aeration and sand filtration stages could further reduce COD values. The Effluent indicators of wastewater quality for irrigation, such as SI, SAR, PI, MR, and MH show relatively safe parameters, although the RSC and SSP exhibit somewhat high values. Reusing and recycling tertiary treated wastewater in agriculture can promote ecological friendliness by avoiding negative effects on aquatic ecosystems and achieving environmental sustainability, even when it involves managing soil quality at the field level. Recommending performing routine maintenance, particularly on the filter media and filtration tanks is essential to increase the degree of environmental awareness among the farmers in the study area. The Arab El-Madabegh WWTP should be expanded to manage the significant daily wastewater loading. This will enhance the effluent quality and prevent the unregulated sewage release. Decision-makers should provide more information on the reuse of treated wastewater as a reliable irrigation source to bridge the growing gap between water demand and supply and prevent wastewater discharge into the environment from contaminating aquatic environments. Consideration of recent data limitations is essential, and future studies should incorporate additional years of observations to understand long-term effects. Further investigation is required to determine the bacterial and harmful components in the plant’s treated wastewater proposed for irrigation, validating and considering these recommendations in future endeavors.

## Supporting information

S1 File (DOCX)
