# Supplementary material for: Comprehensive environmental impact assessment and irrigation wastewater suitability of the Arab El-Madabegh wastewater treatment plant, ASSIUT CITY, EGYPT
Source: PLoS One. 2024 Feb 29;19(2):e0297556. doi: 10.1371/journal.pone.0297556 (PMC10903899; doi:10.1371/journal.pone.0297556)
Supplement: S1 File — (DOCX) [file pone.0297556.s001.docx]

**Supplementary Materials**

**Table S1** Water quality parameters, units and analytical methods (WEF, 2005)

| **Parameter** | **Notation** | **Units** | **Analytical methods** | |
| --- | --- | --- | --- | --- |
| pH | pH | Unit | Using Ultra meter | |
| Ammonia-Nitrogen | NH_3_-N | mg/L | Phenate method | |
| Temperature | Temp. | ◦ C | Using Ultra meter | |
| Nitrite-Nitrogen | NO_2_-N | mg/L | Colorimetric method | |
| Conductivity | EC | ms/cm | Using Ultra meter | |
| Nitrate-Nitrogen | NO_3_-N | mg/L | UV spectrophotometer | |
| Sulfate | SO_4_^2-^ | mg/L | Turbidimetric method | |
| Phosphate | PO_4_^3-^-P | mg/L | Vanadomolybdophosphoric acid colorimetric method | |
| Fluoride | F^-^ | mg/L | SPADNS method | |
| Total Dissolved Solids | TDS | mg/L | Gravimetric method | |
| Chemical Oxygen Demand | COD | mg/L | Open reflux method | |
| Total Organic Carbon | TOC | mg/L | TOC analyzer | |
| Biological Oxygen Demand | BOD | mg/L | 5-day BOD test | |
| Total Suspended Solids | TSS | mg/L | Gravimetric method | |
| Chloride | Cl^-^ | mg/L | Argentometric method | |
| Carbonate | CO_3_^2-^ | mg/L | Titrimetric method | |
| Bicarbonate | HCO_3_^-^ | mg/L | Calculation method | |
| Calcium | Ca^2+^ | mg/L | Titrimetric method | |
| Sodium | Na^+^ | mg/L | Flame photometry | |
| Magnesium | Mg^2+^ | mg/L | Calculation method | |
| Cadmium | Cd | µg/L | GFAAS | |
| Potassium | K^+^ | mg/L | Flame photometry | |
| Lead | Pb | µg/L | GFAAS | |
| Copper | Cu | µg/L | GFAAS | |
| Nickel | Ni | µg/L | GFAAS | |
| Iron | Fe | µg/L | GFAAS | |
| Zinc | Zn | µg/L | GFAAS | |
| Chromium | Cr | µg/L | GFAAS | |
| Manganese | Mn | µg/L | GFAAS | |
| **GFAAS:** graphite furnace atomic absorption spectrophotometer | | | |  |

**Table S2** Maximum discharge limits for treated wastewater into the aquatic environment (MWRI 2009)

| **Parameter** | | **Unit** | **Permissible limit** | | |
| --- | --- | --- | --- | --- | --- |
| pH | | Unit | 6-9 | | |
| Turbidity | | NTU | - | | |
| Temperature | | º C | 35 | | |
| Electrical conductivity | | mS/Cm | - | | |
| Total Dissolved Solids | | mg/L | 2000 | | |
| Total suspended solids | | mg/L | 50 | | |
| Chemical oxygen demand | | mg/L | 80 | | |
| Biological oxygen demand | | mg/L | 60 | | |
| Total organic carbon | | mg/L | - | | |
| Phosphate | | mg/L | 2 | | |
| **Table 2.1:** Continued | | | | |  |
| **Parameter** | **Unit** | | | **Permissible limit** |  |
| Nitrate | mg/L | | | - |  |
| Calcium | mg/L | | | - |  |
| Chloride | mg/L | | | - |  |
| Sulfate | mg/L | | | - |  |
| Carbonate | mg/L | | | - |  |
| Magnesium | mg/L | | | - |  |
| Calcium | mg/L | | | - |  |
| Potassium | mg/L | | | - |  |
| Manganese | mg/L | | | - |  |
| Fluoride | mg/L | | | - |  |
| Sodium | mg/L | | | - |  |
| Cadmium | mg/L | | | 0.003 |  |
| Manganese | mg/L | | | - |  |
| Bicarbonate | mg/L | | | - |  |
| Iron | mg/L | | | - |  |
| Lead | mg/L | | | 0.001 |  |
| Zinc | mg/L | | | 1 |  |
| Nickel | mg/L | | | 0.02 |  |
| Copper | mg/L | | | 1 |  |
| Chromium | mg/L | | | 0.01 |  |

(-) Indicates that there is no a guideline for the parameter

Table S3 Classification of irrigation water based on EC values (Mills, 2003; Singh et al., 2008)

| **Salinity** | **EC** | **Class** |
| --- | --- | --- |
| Low salinity water, suitable for use on all crops except tobacco | < 650 | Class I |
| Medium salinity, suitable for use on all but very low salt tolerant crops | 650-1300 | Class II |
| High salinity, suitable for use on medium and high salt tolerant crops | 1300-3000 | Class III |
| Very high salinity, suitable for use only on high salt tolerant crops | 3000-5000 | Class IV |
| Extremely high salinity generally unsuitable for  irrigation unless soils are permeable | 5000-8000 | Class V |
| Too saline for irrigation | > 8000 | Class VI |

Table S4 Water Quality classification based on SSP according to (Todd and Mays 2004)

| \| **SSP** \| **Quality of water** \| \| --- \| --- \| \| < 20 % \| Excellent \| \| 20-40 % \| Good \| \| 40-60 % \| Permissible \| \| 60-80 % \| Doubtful \| \| > 80 % \| Unsuitable \| |
| --- | --- | --- | --- | --- | --- | --- | --- | --- | --- | --- | --- | --- |

Table S5 Classification of irrigation water based on SAR values (Mills, 2003; Singh et al., 2008)

| **Sodicity class** | **SAR** | **Class** |
| --- | --- | --- |
| No sodium problem | < 3 | Class I |
| Low sodium, few problems except with sodium sensitive crops | 3-6 | Class II |
| Medium sodium, increasing problems | 6-8 | Class III |
| High sodium, not generally recommended | 8-14 | Class IV |
| Very high sodium, unsuitable | > 14 | Class V |

Table S6 Limits of residual Mg/Ca ratio in irrigation water (Paliwal, 1972)

| Quality | MR |
| --- | --- |
| Safe | < 1.5 |
| Moderate | 1.5-3.0 |
| Unsafe | > 3.0 |

Table S7 Classification of irrigation water based on RSC values (Haygarth and Jarvis, 2002)

| Hazard | RSC |
| --- | --- |
| None | < 0 |
| Low, with some removal of calcium and magnesium from irrigation water | 0-1.25 |
| Medium, with appreciable removal of calcium and magnesium from irrigation water | 1.25-2.5 |
| High, with most calcium and magnesium removed leaving sodium to accumulate | > 2.5 |

**References**

Haygarth, P.M., Jarvis, S.C., 2002. Agriculture, hydrology, and water quality. CABI Wallingford.

Mills, B., 2003. Interpreting water analysis for crop and pasture. File No. FS0334, DPI’s Agency Food Fiber Sci. Toowoomba.

Paliwal, K. V, 1972. Irrigation with saline water.

Singh, A.K., Mondal, G.C., Kumar, S., Singh, T.B., Tewary, B.K., Sinha, A., 2008. Major ion chemistry, weathering processes and water quality assessment in upper catchment of Damodar River basin, India. Environ. Geol. 54, 745–758.

WEF, A.A., 2005. Standard methods for the examination of waterand wastewater. Am. Public Heal. Assoc. Am. Work. Assoc. Water Environ. Fed. 21st Ed. Washingt. DC, USA.
